# A detailed characterisation of the distribution and presentation of DNA vaccine encoded antigen

**DOI:** 10.1016/j.vaccine.2009.11.014

**Published:** 2010-02-10

**Authors:** Catherine M. Rush, Timothy J. Mitchell, Paul Garside

**Affiliations:** a Centre for Biophotonics, Strathclyde Institute of Pharmacy and Biomedical Sciences, University of Strathclyde, 27 Taylor St, Glasgow G4 0NR, UK; b Division of Infection and Immunity, Faculty of Biomedical and Life Sciences, University of Glasgow, Glasgow G12 8QQ, UK

**Keywords:** BLN, brachial lymph nodes, CLN, cervical lymph nodes, DLN, draining lymph nodes, eGFP, enhanced green fluorescent protein, ILN, inguinal lymph nodes, PB, peripheral blood, pDNA, plasmid DNA, pMHC, peptide/MHC, Vaccination, Antigen presentation, T cells

## Abstract

The association between plasmid DNA distribution, the amount of Ag produced, Ag persistence and the identity and localisation of cells presenting DNA-encoded Ag all have important consequences for both quantitative and qualitative aspects of T cell responses induced by DNA vaccines. Using a variety of approaches to detect and quantify the uptake of injected DNA, and the production and presentation of DNA-encoded antigen, we report that injected DNA vaccines rapidly enter the peripheral blood from the injection site and also reach muscle-draining lymph nodes directly as free DNA. 24 h after plasmid injection, MHCII^+^CD11b^+^B220^−^CD11c^low/−^ cells in the draining and distal LNs and spleen contain pDNA. Interestingly, we also observed pDNA^+^MHCII^low/−^CD11b^+^ within the bone marrow. Concomitantly, we detected Ag-containing/expressing cells at both the injection site and in draining lymph nodes. Three days after plasmid injection we detected rare pMHC^+^CD11c^+^ cells within secondary lymphoid tissue and simultaneously observed Ag-specific CD4^+^ T cell accumulation and blastogenesis in these tissues. Our results show that the events that determine the induction of DNA vaccine immune responses occur within days of DNA injection and that the response becomes systemic very rapidly, possibly with involvement from resident BM cells.

## 1 Introduction

Studies using protein have shown that the dose and duration of Ag exposure can influence a number of important parameters involved in T cell priming [1–4] including the acquisition of effector functions (e.g. Th1/Th2 phenotype) [5–7], memory cell differentiation and the size of the memory cell pool [8,9]. Thus, the relationships between Ag dose and distribution, the number of pMHC complexes on an APC, costimulatory molecule interactions and pMHC/TcR stability determine the nature and extent of T cell activation and function.

Due to their non-replicative nature, DNA vaccines produce very low amounts of antigen *in vivo* (nanogram range), even when using the strongest viral promoters to drive Ag production [10]. However, despite the low amounts of Ag involved, and although primary immune responses can be difficult to demonstrate [11], recall responses are often potent [11]. This may be related to the fact that, in contrast to other immunisation strategies where large bolus doses of Ag are administered, DNA vaccines are characterised by sustained production of small amounts of Ag [10]. Hence the links between pDNA distribution following injection, amount of Ag produced (and Ag persistence) and the identity and localisation of cells presenting DNA-encoded Ag may have important consequences for both quantitative and qualitative aspects of T cell responses induced by DNA vaccines. However, the relationship between cells that acquire pDNA, and those expressing or presenting DNA-encoded Ag to naïve T cells is still unclear. Thus, in the context of intramuscular DNA vaccination it will be important to determine the distribution of cell-associated DNA; which cells produce and present antigen; where, when and how long they do this for; their phenotype and activation status and the relationship between these parameters and CD4^+^ T cell activation. To address some of these questions we have used a system based on the Eα_52–68_ peptide of the MHC I-E molecule that allows us to detect Ag, pMHC complexes and Eα-specific T cells directly in tissue [1].

Our results show that the events that determine the induction of DNA vaccine immune responses occur within hours/days of DNA injection and that the response becomes systemic very rapidly, possibly with involvement from resident BM cells. Such understanding of the anatomical location, kinetics and cellular mechanisms influencing the development and maintenance of DNA vaccine-induced immune responses may be important for fully exploiting their potential by allowing rational design.

## 2 Materials and methods

### 2.1 Mice

CD4 T cells from TEa mice recognise the I-E-derived peptide E alpha 52–68 (Eα_52–68_) in the context of I-A^b^ [12]. TEa mice expressing the Thy1.1 allele were obtained from S. McSorley (University of Minnesota, Minneapolis, MN) and used as Tg CD4 T cell donors. C57 BL/6 (B6) (Thy1.2, Ly5.2) mice were purchased from Harlan UK Ltd. (Bicester, UK). Animals were maintained at the Central Research Facility (University of Glasgow, Glasgow, UK) under specific pathogen free conditions and all procedures performed according to local and UK Home Office regulations. Male and female mice aged 6–12 weeks were used in all experiments.

### 2.2 Antibodies and reagents

The mouse monoclonal Ab Y-Ae (murine IgG2b) has been described previously [1,3,13]. Y-Ae recognises the Eα_52–68_ peptide in the context of the I-A^b^ MHC Class II molecule [3,13]. Biotinylated Y-Ae was prepared in-house using the Y-Ae hybridoma kindly provided by S. McSorley (University of Minnesota). Biotinylated isotype control mouse IgG2b was from Southern Biotechnology. Hamster anti-CD11c (N418) and hamster IgG isotype were from Serotec. Biotinylated goat anti-rabbit IgG and goat anti-hamster IgG were from Vector Laboratories Ltd. Rabbit anti-GFP IgG, Streptavidin-Alexa Fluor 647 (SA-AF647), Avidin-Cascade Blue and Alexa Fluor dye tyramide kits were from Molecular Probes (Invitrogen). Biotinyl tyramide signal amplification kits were from PerkinElmer. The following fluorochrome-conjugated and biotinylated antibodies were from BD Pharmingen: anti-CD4/L3T4 (GK1.5 and RM4-5), anti-CD69 (H1.2F3), anti-CD45R/B220 (RA3-6B2), anti-CD11c (HL3), anti-CD11b (M1/70), anti-I-A/I-E (2G9), anti-Vβ6 (RR4.7), anti-Vα2 (B20.1), and anti-Ly5.2 (104). Streptavidin-APC (SA-APC) was from BD Pharmingen.

### 2.3 Generation of EαGFP fusion protein and protein purification

The *Escherichia coli* strain expressing the EαRFP fusion protein has been described previously [1] and was kindly provided by M.K. Jenkins and S. McSorley (University of Minnesota). This protein is encoded by an in-frame fusion between amino acids 45 and 73 of the MHC Class II I-E molecule (containing Eα_52–68_) and the Red Fluorescent Protein, DsRed1 (Clontec). We constructed an alternative version of this protein in pTrcHisTOPO (Invitrogen) by replacing the RFP coding sequence with the eGFP coding sequence from pEGFP-N1 (Clontech), to generate an EαGFP gene fusion (pTrcHisEαGFP). Expression of EαGFP protein was induced using 1 mM IPTG for 16–18 h and protein was purified using Ni-NTA Superflow resin (Qiagen). Purified protein was quantified using Coomassie Plus Protein Assay Reagent (Pierce).

### 2.4 DNA vaccine constructs

The plasmid pCI-EαRFP was prepared by PCR cloning of the EαRFP coding sequence from the previously described plasmid pTrcHisEαRFP [1] into the mammalian expression plasmid pCIneo (Promega). The plasmid pCI-EαGFP was created by PCR using pTrcHisEαGFP as template. The plasmid pCI-OVAeGFP expresses a cytosolic OVAeGFP fusion protein.

### 2.5 Cell lines, transfection and cross-presentation assay

HeLa cells were cultured in DMEM supplemented as described above and were transfected using Lipofectamine 2000 (Invitrogen) according to the manufacturer's instructions. To ensure that pCI-EαGFP- and pCI-EαRFP-expressed EαGFP and EαRFP proteins could be correctly processed and the Eα peptide surface displayed, we set up co-culture, cross-presentation assays using transfected HeLa cells as a source of Eα antigen and B6 (I-E^−^/I-A^b+^) BMDCs as APCs. HeLa cells (obtained from ECACC) were seeded in chamber slides and transfected with pCI-EαGFP, pCI-EαRFP, or control plasmids pCIneo or pCI-OVAeGFP. 24 h post-transfection, B6 BMDCs prepared as described previously [14], were added and cells were co-cultured to allow DCs to acquire plasmid-expressed Ag. BMDC cultures typically contained 85–90% CD11c^+^ cells. 4 h later, LPS (from *Salmonella equi-abortus*, Sigma) was added to a final concentration of 1 μg/ml to induce DC maturation. After 24 h co-cultured CD11c^+^ DCs were analysed for GFP and surface Y-Ae staining by flow cytometry and by immunofluorescence staining of cells seeded in chamber slides.

### 2.6 Adoptive transfer

Lymph node and spleen cell suspensions from TEa Tg mice were prepared as previously described [1]. The Eα peptide-specific Tg CD4 T cells were identified as CD4^+^Vβ6^+^Vα2^+^. B6 recipients received 0.5–1 × 10^6^ Tg T cells in 0.2 ml intravenously in the lateral tail vein 1 day prior to immunisation. In some experiments Tg T cells were labelled with CFSE prior to adoptive transfer as previously described [15].

### 2.7 Immunisation

For EαGFP protein immunisation, different doses (100 μg, 10 μg, 1 μg, 100 ng, 10 ng and 1 ng) diluted in PBS, were administered subcutaneously in the neck scruff, each with 1 μg/dose LPS (*S. equi-abortus*, Sigma) as adjuvant. Control mice received PBS containing 1 μg LPS. LPS was added in order to activate APC and drive them from an antigen acquisitive to antigen presenting state as widely described in the literature. For intramuscular DNA immunisation mice received 50 μg plasmid DNA diluted in endotoxin-free PBS in a 50 μl final volume in both *tibialis anterior* (TA) muscles.

### 2.8 Preparation of cells for flow cytometry

At various times after EαGFP subcutaneous protein immunisation and subcutaneous DNA injection, cervical (CLN), brachial (BLN) and inguinal (ILN) lymph nodes were removed, macerated through Nitex mesh (Cadish and Sons, London, UK) and digested with 1 mg/ml Collagenase A (Sigma) and 10 μg/ml DNase A (Roche Diagnostics) in HBSS for 30 min at 37 °C. EDTA was added to a final concentration of 10 mM and cells incubated a further 5 min at room temperature to maximise the number of dendritic cells released from tissues. Popliteal and inguinal lymph nodes that drain the lower limbs, were removed at various times after intramuscular DNA injection and single cell suspensions prepared as described above. GFP^+^ cells were identified in the FL1 channel of the FACsCalibur flow cytometer (Becton Dickinson). Cells displaying Eα peptide–MHC complexes were identified using biotinylated Y-Ae and SA-APC. PE-conjugated anti-CD11c was used to identify dendritic cells. In adoptive transfer experiments, Eα-specific TEa T cells were identified using Alexa Fluor 647-conjugated anti-CD90.1 (Thy1.1) (HIS51) (Serotec) and PE-conjugated anti-CD4. A FacsCalibur flow cytometer was used with CellQuest acquisition software and FlowJo analysis software (Treestar).

### 2.9 Plasmid labelling and detection of labelled DNA *in vivo*

pCIneo or pCI-EαRFP plasmid DNA was labelled using the Label-IT Cy5 kit (Mirus Bio) according to the manufacturer's instructions. 20 μg of labelled plasmid in 50 μl PBS was injected intramuscularly (TA muscle) and at various times after injection, draining popliteal and ILNs, distal CLNs and BLNs, spleens, peripheral blood and bone marrow were collected for flow cytometry. Phenotypic characterisation of cells carrying pDNA-Cy5 was performed using fluorochrome-labelled lineage specific markers including MHC Class II, CD45 (Ly5.2 allotype for B6 mice), CD11b, CD11c and B220.

### 2.10 Detection of Ag and pMHC complexes in tissue sections

At various times after EαGFP (or EαRFP) protein or DNA immunisation, injection sites (skin or muscle) draining and non-draining lymph nodes and spleens were excised and post-fixed in 1% paraformaldehyde (PFA)/PBS for 2 h. Tissues were quenched for 10 min in 0.5% Gly-Gly (Sigma), followed by 2 h in 10% sucrose/PBS, then overnight in 30% sucrose/PBS before embedding in OCT medium (Miles, Elkart, USA) and snap freezing in liquid nitrogen. We found that this fixation procedure preserved GFP fluorescence, which is often liable to diffusion in unfixed tissue, but still preserved conformational epitopes including pMHC complexes. 18–20 μm sections of TA muscles were mounted with Vectashield containing the nuclear stain DAPI (Vector) and examined for GFP fluorescence. Frozen sections of lymph nodes, cut at 6–8 μm were air-dried, rehydrated in PBS, permeabilised in 0.1% Triton X-100/PBS, washed briefly in PBS, treated with 1%H_2_O_2_/0.1% sodium azide/PBS to destroy endogenous peroxidases, and blocked using the Avidin/Biotin blocking kit (Vector) and anti-CD 16/CD32 (BD Pharmingen). The GFP signal in tissue sections was amplified using rabbit anti-GFP IgG, biotinylated goat anti-rabbit IgG, SA-HRP (Tyramide Signal Amplification kit, PerkinElmer), biotinyl tyramide and SA-647 or SA-488. Y-Ae^+^ cells were localised using biotinylated Y-Ae mAb, followed by SA-HRP, biotinyl tyramide and either SA-AF647 or Avidin-Cascade Blue. Control sections were treated as above but were incubated with the Y-Ae isotype, i.e. biotinylated mouse IgG2b. B cell follicles were stained using Pacific Blue-conjugated anti-CD45R/B220 (prepared in-house) or FITC-conjugated anti-CD45R/B220 for EαRFP and pCI-EαRFP samples. CD11c^+^ cells in Y-Ae-stained sections were demonstrated by first staining with Y-Ae as described above, followed by additional H_2_O_2_/azide treatment and avidin and biotin blocking, to remove unreacted HRP and biotin/avidin, respectively. Sections were then incubated in either hamster anti-CD11c or hamster IgG (isotype control), biotinylated goat anti-hamster IgG, SA-HRP and Pacific Blue tyramide. Slides were mounted in Vectashield and images were captured using an Olympus BX-50 microscope with colour CCD digital camera and OpenLab digital imaging software (Improvision, Coventry, UK). In some images fluorochromes were false coloured to improve image colour contrast.

### 2.11 Statistics

Results are expressed as mean ± SE mean when *n* ≥ 3 and mean ± range where *n* = 2. Student's unpaired *t* tests with two-tailed distribution were used to calculate statistical significance (*p* < 0.05) when samples were normally distributed.

## 3 Results

### 3.1 Establishing detection of Ag and pMHC complexes *in vivo* following protein immunisation

Elegant studies by Itano et al. [1] described a novel system for studying Ag distribution, and identifying cells presenting Ag *in vivo*, in conjunction with Ag-specific CD4^+^ T cells recognising the same pMHC complex. We adapted these tools to investigate Ag and APCs in the context of DNA vaccination. The original study [1] utilised an EαRFP (or EαDsRed) fusion protein for Ag detection. As others have reported cytotoxicity and aggregation associated with the DsRed1 protein used in this fusion protein and because we wanted to be able to further amplify the Ag signal, we developed an Ag detection system based on the monomeric eGFP. We modified the system described previously by replacing the RFP(DsRed1)-component with a sequence encoding eGFP and validated the EαGFP system for detection of both Ag and pMHC complexes *in vivo*.

**Fig. 1:**
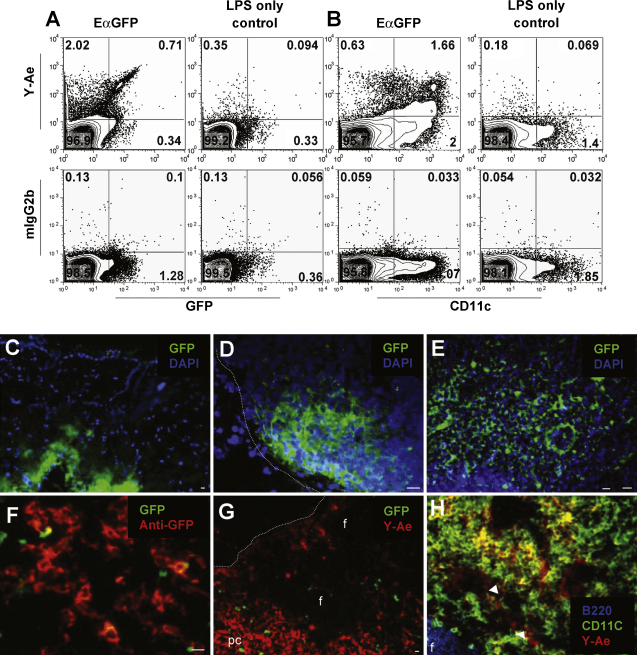
Ag and pMHC complexes were detected in draining lymph nodes following EαGFP immunisation. 24 h after EαGFP injection, flow cytometry was used to assess the proportion of live cells in pooled draining CLNs and BLNs that were GFP^+^, pMHC^+^ (i.e. Y-Ae^+^) and CD11c^+^ (A and B). Control mice received LPS only. Mouse IgG2b was used as Y-Ae isotype control for each sample and was used to set positive and negative quadrant gates. Representative dot plots are shown for each group (*n* = 3). Fluorescence microscopy was used to demonstrate EαGFP (green) in tissue sections of the injection site (C) and in draining CLNs (D and E), 30 min after protein injection. The intrinsic GFP signal was amplified using a GFP-specific polyclonal Ab (red in F). At 24 h post-injection, large areas of the paracortex (pc) of DLNs were Y-Ae^+^ (G), whereas B cell follicle areas (f) were not stained. Phenotypic characterisation using lineage specific Abs (H) showed that many Y-Ae^+^ cells were also CD11c^+^ (yellow), although some Y-Ae^+^ cells did not costain with CD11c (red, indicated by arrows). (H) Fluorochromes were false coloured to improve colour contrast. Dotted lines indicate the periphery of LNs. Representative data is shown. Scale bars show approximately 10 μm. (For interpretation of the references to colour in this figure legend, the reader is referred to the web version of the article.)

Subcutaneous immunisation with EαGFP protein resulted in marked heterogeneity in both Ag content and pMHC complex display in the cells of draining lymph nodes. Flow cytometric analysis of lymph node suspensions from mice immunised 24 h previously with 100 μg EαGFP protein plus 1 μg LPS showed that about 2.3–2.7% of all live cells were Y-Ae^+^ compared to about 0.4% for control mice immunised with LPS alone (Fig. 1A and B, upper panels). The Y-Ae isotype control antibody mIgG2b was used to set positive staining gates and showed approximately 0.2% background staining (Fig. 1A and B, lower panels). Hence, the maximum background Y-Ae staining (LPS and isotype control) is approximately 0.4% and staining above this level is considered positive staining. Background staining could not be completely eliminated due to tissue autofluorescence and the large numbers of cells that were acquired for analysis.

The majority of Y-Ae^+^ cells found in draining lymph nodes at 24 h post-injection were GFP^low/−^ or below the level of GFP detection (∼2.0% of live cells, Fig. 1A, upper left quadrant) with only 0.7% of live cells displaying a Y-Ae^+^GFP^high^ phenotype (Fig. 1A, upper right quadrant). Interestingly, there was considerable heterogeneity in CD11c staining within the Y-Ae^+^ population (Fig. 1B) with several different populations with different levels of Y-Ae staining or CD11c expression clearly evident. In this experiment, approximately 50% of CD11c^high^ cells from EαGFP-immunised mice were Y-Ae^+^ (Fig. 1B, upper panel, upper right quadrant), however, there were a smaller percentage (∼28%; ∼0.6% of live cells) with a Y-Ae^+^CD11c^low/−^ phenotype (Fig. 1B, upper panel, upper left quadrant). At present we have not attempted to further characterise these Y-Ae^+^CD11c^low/−^ cells.

EαGFP Ag was demonstrated at both the injection site (Fig. 1C) and in the local draining lymph nodes (Fig. 1D and E) 30 min after injection. EαGFP appeared to flow from one side of the lymph node, from the subcapsular sinus into the paracortical areas (Fig. 1E) as has been observed previously for other protein Ags, including EαRFP [1]. To maximise the sensitivity of Ag detection in lymphoid tissues, we used GFP-specific rabbit IgG to amplify the GFP signal (Fig. 1F). At 24 h we observed that large areas of the draining lymph nodes were Y-Ae^+^ (Fig. 1G) as has been reported previously [1]. B cell follicular areas were not stained with Y-Ae, with the majority of Y-Ae^+^ cells being found in the interfollicular areas, paracortex and subcapsular sinus. As was observed by flow cytometry, Y-Ae staining co-localised with CD11c^+^ cells (Fig. 1H, yellow), however there were some Y-Ae^+^CD11c^low/−^ cells (red).

### 3.2 Dose-dependent detection of Ag and pMHC complexes following protein immunisation

The maximum amount of Ag detected following DNA vaccination is known to be in the nanogram range in muscle and serum [10,16], however the amount of Ag that reaches lymphoid tissues is unknown. Estimates are that fewer than 2% of all CD11c^+^ cells may contain plasmid-encoded Ag following transdermal gene gun delivery [17] and it is not known how many of these cells present Ag to naïve lymphocytes. Therefore we wished to establish sensitive methodologies to study those cells that acquire and present DNA-encoded Ag, particularly in lymphoid tissue. To determine the minimum amount of protein Ag that could be detected *in vivo* and how much Ag is needed to be able to detect cells displaying pMHC complexes, we administered a range of doses of EαGFP protein and examined the draining lymph nodes for cell-associated Ag and cells displaying pMHC complexes. The aim of this protein injection study was to demonstrate the sensitivity of the assay systems in a widely studied situation such as subcutaneous injection.

**Fig. 2:**
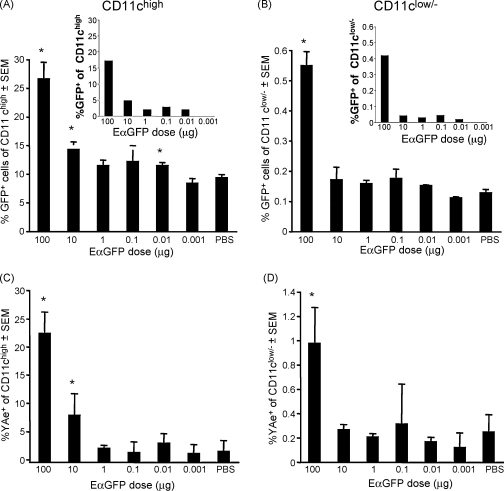
Dose-related detection of GFP-containing and pMHC^+^ cells in peripheral lymph nodes 24 h after Ag injection. Different doses of EαGFP Ag (100 μg, 1 μg, 0.1 μg, 0.01 μg, 0.001 μg) each containing 1 μg LPS were administered by subcutaneous injection in the neck scruff. Mice immunised with PBS containing 1 μg LPS were used for controls to establish the baseline staining and assay sensitivity. Draining proximal CLNs and BLNs were collected at 24 h from individual mice. The proportion of GFP^+^ cells in gated CD11c^high^ (A) and CD11c^low/−^ (B) populations was determined for each sample by flow cytometry and results expressed as group mean percentage ± standard error of the mean (SEM). The high proportion of apparently GFP^+^ CD11c^+^ cells in the PBS/LPS control (∼10–15%) is due to background autofluorescence in the CD11c^+^ gate resulting from the collection of the large numbers of cells needed for analysis of the small fraction (1–4%) of lymph node CD11c^+^ cells. If the mean autofluorescence of the PBS control group is subtracted (insets in A and B) from the mean percentage of the groups receiving 0.001–100 μg EαGFP, it is clear that GFP^+^ cells are found in draining LNs for all antigen doses ranging from 100 μg to 10 ng. The proportion of CD11c^high^ and CD11c^low/−^ cells displaying pMHC complexes is shown in (C) and (D), respectively. Mouse IgG2b was used as the Y-Ae isotype control for each sample and used to set positive and negative gates. Error bars show SEM for each group, where *n* = 3. Statistical comparisons (Students’ unpaired *t*-test, 2 way, assuming unequal variance) were made between the PBS control group and each dose and asterisk (*) indicates statistical significance where *p* < 0.05.

Both Ag distribution and the proportion of GFP^+^ cells were influenced by Ag dose (Fig. 2A and B). GFP^+^ cells were detected in the CLNs (Fig. 2A and B), BLNs and ILNs (data not shown), 24 h after injection of 100 μg Ag (*n* = 3, *p* < 0.05). However, lower Ag doses yielded far fewer GFP^+^ within both the CD11c^+^ (Fig. 2A) and CD11c^low/−^ (Fig. 2B) populations. Statistical significance was only demonstrated for 100 μg, 10 μg and 10 ng doses, as often 1 out of 3 mice showed significant variability from the other 2 mice. Although the percentage of GFP^+^ cells in the CD11c^low/−^ population following 10 μg, 1 μg and 0.1 μg Ag doses appeared elevated compared to PBS/LPS control, particularly in draining CLN and BLN, these were not statistically significant. The proportion of CD11c^low/−^ cells containing GFP following 100 μg Ag, was higher in the local cervical and brachial LNs than in more distal inguinal and axial LNs (data not shown). Background correction, calculated by subtracting mean values for PBS control from dose values revealed that GFP^+^ cells could be detected at low Ag doses (Fig. 2A and B, insets). The amount of cell-associated GFP from doses less than 100 μg may be below the level of sensitivity of GFP detection by flow cytometry. Lymphoid tissue autofluorescence also impacts on assay sensitivity.

Analysis of cells displaying pMHC complexes (i.e. Y-Ae^+^) revealed that we could detect complexes in more than 20% of all CD11c^high^ cells in the draining CLNs (Fig. 2C) and BLNs (not shown) at the 100 μg dose. Decreasing amounts of Ag resulted in corresponding decreases in the percentages of CD11c^+^Y-Ae^+^ cells, with the limit of detection of pMHC complexes between 1 μg and 100 ng of administered Ag. pMHC complex detection in CD 11c^low/−^ cells showed a similar trend. As was the case for detection of GFP^+^ cells, variability within the small group (*n* = 3), limited statistical significance. Both the CD11c^high^ and CD11c^low/−^ populations also showed increased, although not statistically significant, Y-Ae mean fluorescence down to a dose of 100–10 ng Ag (data not shown).

These results indicate that with controlled and careful detailed analyses, we can detect both Ag and cells displaying pMHC complexes following administration of about 1 μg–100 ng Ag, and this is the upper limit of Ag that we might expect to be produced following pDNA injection.

### 3.3 Kinetics of Ag distribution and pMHC complex formation following protein immunisation

**Fig. 3:**
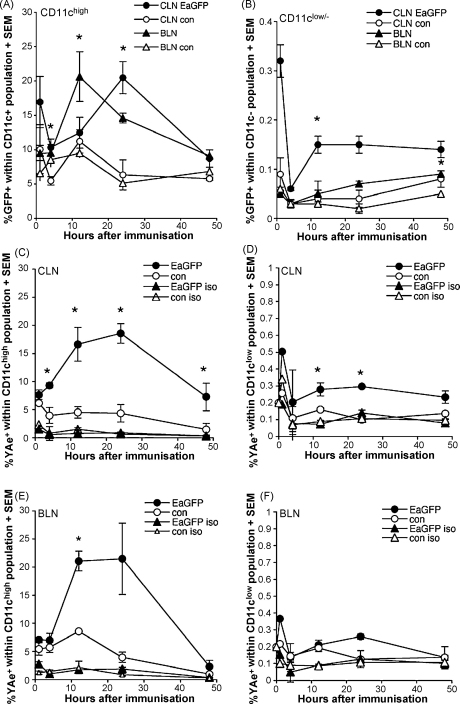
Kinetics of appearance of EαGFP and pMHC positive cells in lymph nodes draining the injection site. Flow cytometry was used to assess the proportion of CD11c^high^ and CD11c^low/−^ cells carrying EαGFP (A and B) and displaying pMHC complexes (C–F) at 1 h, 4 h, 12 h, 24 h and 48 h after EαGFP/LPS injection. Cell suspensions were prepared from the CLNs and BLNs of mice receiving either 100 μg EαGFP/LPS or PBS/LPS alone. Live cells were gated for CD11c expression and CD11c^high^ and CD11c^low/−^ gated cells were analysed separately for both GFP and Y-Ae staining. The mean percentage of CD11c^+^ cells in CLNs (closed circles, ●) and BLNs (closed triangles, ▴), that are GFP^+^ at each timepoint, are shown in (A). Open circles (○) and open triangles (▵) represent background staining in CLNs and BLNs of control mice receiving PBS/LPS only. Results for CD11c^low/−^ cells are shown in (B). The mean percentage of CD11c^+^ cells in CLNs, at each timepoint, that are surface Y-Ae^+^ following EαGFP (●) or PBS/LPS control immunisation (○) are shown in (C). Mouse IgG2b was used as the Y-Ae isotype control to set positive and negative gates and is represented as open symbols in (C). (D) Results for Y-Ae staining in CD11c^low/−^ cells. Kinetic results for CD11c^high^ and CD11c^low/−^ cells in BLN are shown in (E) and (F), respectively. Statistical comparisons were made between the EαGFP/LPS group (*n* = 3) and control PBS/LPS group (*n* = 3) for each timepoint and asterisk (*) indicates significance where *p* < 0.05 using the Student's *t*-test. Error bars show standard error of the means (SEM).

The kinetics of Ag distribution and presentation is likely to vary depending on the route (e.g. subcutaneous vs. intramuscular) and the type of immunisation (e.g. protein vs. pDNA), and we wished to determine the kinetics of appearance of pMHC complexes for both protein and pDNA immunisation. The aim of this protein injection study was to study the kinetics of Ag distribution in a widely studied situation such as subcutaneous injection. As has been shown for EαRFP previously [1], EαGFP^+^ cells, i.e. cell-associated EαGFP, can be found in the neck-draining CLNs and BLNs within 1 h of Ag injection in both CD11c^high^ (Fig. 3A) and CD11c^low/−^ (Fig. 3B) cells. Fluorescence microscopy indicated that in addition to this cell-associated Ag, much of the injected Ag appeared to be extracellular (Fig. 1D). After this initial wave of antigen positive cells in the draining LNs, the number of cells carrying or associated with Ag decreased until 12–24 h when GFP^+^ cells reappeared in draining LNs. CD11c^+^GFP^+^ cells reappeared in the BLNs prior to their reappearance in the CLNs (Fig. 3A). Itano et al. [1] suggested that these Ag-bearing cells have migrated from the injection site. Although many of these LN immigrants are likely to be dendritic cells, some CD11c^low/−^ cells also appeared in the LN at this timepoint (Fig. 3B). We have not attempted to further characterise these cells. Following the initial peak in immigration into the LNs, numbers of GFP^+^CD11c^+^ and GFP^+^CD11c^low/−^ cells gradually declined over the next 24 h and we were still able to detect GFP^+^ cells at 48 h (Fig. 3A and B) and low numbers 3–7 days after immunisation (data not shown). In all cases results were compared to control mice that had received LPS only and showed only minimal background staining.

The appearance of Y-Ae^+^ cells in both the CLNs and BLNs, showed similar kinetics to that of GFP^+^ cells, with small numbers of CD11c^high^ and CD11c^low/−^ displaying pMHC complexes as early as 1 h after Ag injection (Fig. 3C–F). The CLNs (Fig. 3C and D) and BLNs (Fig. 3E and F) showed similar numbers of Y-Ae^+^ cells at the timepoints examined, although statistical analysis revealed that the %Y-Ae^+^ cells in CLNs were statistically higher than controls at a number of timepoints whereas %Y-Ae^+^ cells in BLNs were significantly above controls at only the 12 h timepoint. By 4 h post-injection there were significantly more Y-Ae^+^CD11c^+^ cells in the CLN compared to the LPS only control (Fig. 3A). Minimal staining with the isotype control mIgG2b antibody confirmed the specificity of the Y-Ae staining. The proportion of draining LN (CLN and BLN) CD11c^+^ and CD11c^low/−^ cells displaying pMHC complexes peaked between 12 and 24 h after immunisation and then decreased by 48 h. In other experiments we were still able to detect pMHC^+^ cells more than 5 days after immunisation (data not shown). Both GFP^+^ and Y-Ae^+^ cells were detected in more distal lymph nodes, including the inguinal and axial LNs, although the proportion and mean fluorescence was lower than in the LNs directly draining the injection site (data not shown).

### 3.4 Expression of Ag and pMHC from DNA vaccine constructs *in vitro*

**Fig. 4:**
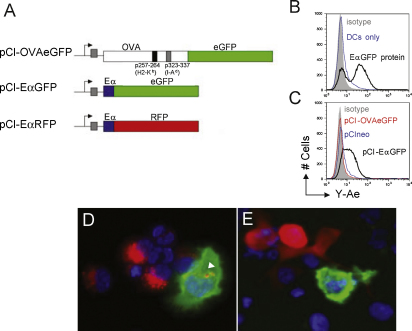
DNA vaccine expression constructs used in this study. (A) Constructs were all based on the pCIneo (Promega). pCI-CytOVAeGFP encodes a cytosolic form of an OVAeGFP fusion protein for simultaneous detection of OVA and eGFP *in vivo*. pCI-EαGFP encodes the EαGFP fusion protein described above and is used for identifying cells expressing (GFP) and presenting (Eα) the Ag EαGFP. pCI-EαRFP is similar to pEαGFP except for the substitution of RFP. (B–E) *In vitro* Ag presentation assay to demonstrate that plasmid-expressed EαGFP and EαRFP is processed and the Eα peptide displayed on the surface of APCs *in vitro*. HeLa cells were transfected with the plasmid constructs pCIneo, pCI-OVAeGFP, pCI-EαGFP, or pCI-EαRFP and B6 bone marrow DCs (+LPS) were added and cells were co-cultured for 24 h to allow DCs to acquire, process and display plasmid-expressed Ag. For positive controls, HeLa/DC co-cultures were pulsed with EαGFP or EαRFP protein. Cells were harvested, stained for CD11c and pMHC complexes using Y-Ae or isotype control (mIgG2b) and analysed by flow cytometry. (B) DCs pulsed with EαGFP were Y-Ae^+^ (surface Eα peptide:MHC ClassII complex) (black), whereas both unpulsed DCs (blue) and isotype controls (grey shading) show minimal staining. (C) Y-Ae^+^ DCs were only present when DCs were co-cultured with pCI-EαGFP-transfectants (black) but not with pCIneo (blue) nor pCI-CytOVAeGFP (red) control transfectants. Isotype controls showed little staining (grey shading). Flow cytometry results for pCI-EαRFP were similar to those for pCI-EαGFP and are not shown. (D) Immunofluorescence staining of cytospins of EαRFP protein-pulsed HeLa/DCs shows, EαRFP-containing monocytic cells (red) with a larger dendritic-like cell also staining with Y-Ae mAb (green). Intact EαRFP Ag is also visible within the Y-Ae^+^ cell (indicated by arrow). (E) Y-Ae^+^ cells are also present in pCI-EαRFP-transfected HeLa/DC cultures (green). A transfected HeLa cell (red) can be seen adjacent to the Y-Ae^+^ cell. (For interpretation of the references to colour in this figure legend, the reader is referred to the web version of the article.)

Before using pCI-EαGFP and pCI-EαRFP DNA vaccine constructs (Fig. 4A) for detection of Ag and pMHC complexes *in vivo*, we wanted to confirm that pCI-EαGFP- and pCI-EαRFP-expressed EαGFP and EαRFP proteins could be correctly processed and the Eα peptide surface displayed on APCs. However because the transfection efficiency of primary DCs, particularly by non-viral vectors is relatively low [18], we established a co-culture assay using transfected HeLa cells as an Ag source and B6 (I-E^−^/I-A^b+^) BMDCs as APCs. In this cross-presentation assay, Ag is transferred to the DCs and processed for peptide presentation in complex with I-A^b^. Hence, positive Y-Ae staining on DCs would indicate the presence of plasmid-derived Eα peptide. HeLa cells were transfected with the plasmid constructs pCI-EαGFP, or pCI-EαRFP or the control constructs pCIneo or pCI-OVAeGFP. 24 h post-transfection, B6 BMDCs were added and cells were co-cultured for 24 h to allow DCs to acquire, process and display plasmid-expressed Ag. For positive controls, HeLa/DC co-cultures were pulsed with EαGFP or EαRFP protein for 16 h. Cells were harvested, stained for CD11c and Y-Ae or CD11c and the Y-Ae isotype control (mouse IgG2b) and analysed by flow cytometry. DCs pulsed with EαGFP were Y-Ae^+^ (surface Eα peptide:MHC ClassII complex) (Fig. 4B, black histogram), whereas both unpulsed DCs (blue histogram) and isotype controls (grey shading) show minimal staining. Flow cytometric analysis of CD11c^+^ cells from plasmid-transfected HeLa/DC cultures, revealed Y-Ae^+^ DCs when DCs were co-cultured with pCI-EαGFP-transfectants (Fig. 4C, black histogram) but not with pCIneo (blue histogram) or pCI-OVAeGFP (red histogram) control transfectants. Isotype controls showed little staining (grey shading). Flow cytometry results for pCI-EαRFP were similar to those for pCI-EαGFP and are not shown. Immunofluorescence staining of EαRFP protein-pulsed HeLa/DCs grown in chamber slides, clearly demonstrated the presence of both Ag-laden cells (red) and pMHC^+^ (Y-Ae^+^) cells (green) (Fig. 4D). Some unprocessed EαRFP can be seen in the cytosol of the Y-Ae^+^ cell (indicated by arrow). We also demonstrated pMHC^+^ cells (green) in pCI-EαRFP-transfected HeLa monolayers co-cultured with BMDCs (Fig. 4E). In this example pCI-EαRFP-transfected HeLa cells expressing the EαRFP protein (red) can be seen adjacent to a Y-Ae^+^ cell (green), suggesting that the Y-Ae^+^ cell had acquired Ag or Eα peptide from another cell (i.e. cross-presentation). These results indicate that our Eα-based DNA vaccine constructs, in combination with the pMHC Ab Y-Ae, may be useful tools for identifying cells presenting DNA-encoded Ag *in vivo*.

### 3.5 pDNA distribution following plasmid injection

**Fig. 5:**
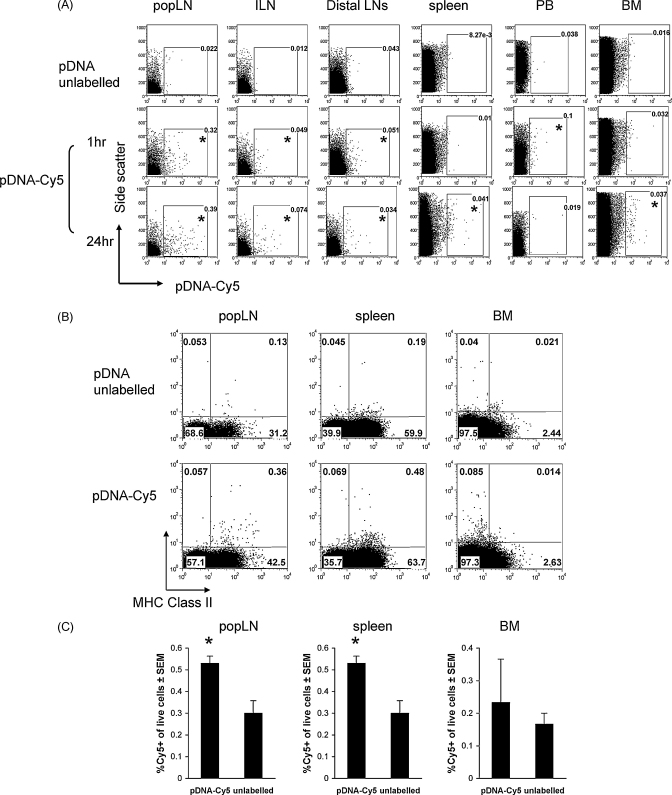
pDNA distribution and phenotype of pDNA-containing cells following plasmid injection. (A) Tissues including the TA muscle, draining popliteal (popLN) and inguinal lymph nodes (ILN), distal brachial and cervical lymph nodes (distal LNs), spleen, peripheral blood and bone marrow were collected 1 h and 24 h following intramuscular injection of Cy5-labelled (pDNA-Cy5) or unlabelled control pDNA. Cell suspensions were examined for the presence pDNA-Cy5 by flow cytometry. Live cell gates (determined by forward and side scatter characteristics) were drawn and cell suspensions from mice receiving unlabelled pDNA were used to set cutoffs for positive and negative gates and represented background tissue staining. Cy5 fluorescence was assessed using the FL4 channel of the FACScalibur and cells with above background fluorescence were considered positive. (A) Cy5 fluorescence (X-axis) vs. side scatter (Y-axis) for representative samples from various tissues at 2 different timepoints. At 1 h we observed pDNA-Cy5 in popliteal, inguinal and distal peripheral lymph nodes with largest numbers in the local muscle-draining popliteal LN. We also observed a few pDNA-Cy5^+^ cells in peripheral blood, but none were detected in either spleen or bone marrow. Asterisk (*) indicates tissues with Cy5^+^ cells. At 24 h post-pDNA-Cy5 injection we found Cy5^+^-cells in draining and distal peripheral lymph nodes. The popliteal LN contained the highest percentage of positive cells. Although we were unable to find pDNA-containing cells in the peripheral blood at 24 h, we were able to demonstrate positive cells in both the spleen and BM. Further characterisation of cell suspensions from pDNA-Cy5 immunised mice shown that pDNA-Cy5^+^ cells from the popLN and spleen were MHCII^+^, whereas those from BM were predominantly MHCII^−^ (B). Statistical comparisons (Students’ unpaired *t*-test, 2 way, assuming unequal variance) were made between the percentage of Cy5^+^-live cells in pDNA-Cy5 injected mice and background staining from unlabelled mice. (C) The mean percentage of Cy5^+^ cells in popLN, spleen and BM. Asterisk (*) indicates statistical significance where *p* < 0.05 (*n* = 3).

We prepared fluorescently labelled plasmid according to standard protocols, injected labelled plasmid and attempted to identify its distribution and the phenotype of associated cells. Tissues including the TA muscle, draining popliteal and inguinal LNs, distal cervical and brachial LNs, spleen, peripheral blood and bone marrow, were collected 1 h and 24 h after intramuscular injection of Cy5-labelled plasmid (pDNA-Cy5) or unlabelled control plasmid (pDNA). Cell suspensions and tissue sections were examined for the presence pDNA-Cy5 by flow cytometry and fluorescence microscopy (data not shown), respectively. We detected extensive Cy5^+^ signal in muscle 1 h after injection using fluorescence microscopy (data not shown). The signal was predominantly between muscle bundles and within myocytes, as has been shown by others previously [19]. During the preparation of the labelled pDNA we removed any unbound Cy5 by extensive washing and thus we are confident that Cy5 signal distribution corresponds with pDNA distribution. 1 h post-pDNA-Cy5 injection, we observed cell-associated pDNA-Cy5 in popliteal, inguinal and distal peripheral LNs by flow cytometry with the largest numbers found in the local muscle-draining popliteal LNs (Fig. 5A, middle panel, asterisks show samples with cell-associated pDNA-Cy5). Cell suspensions from the different tissues of individual mice (*n* = 3 mice per group for each timepoint) were gated on live cells (based on forward and side scatter plots) and positive and negative gates were set using cell suspensions from equivalent tissues collected from mice injected with unlabelled pDNA (Fig. 5A, top panel). We observed a few pDNA-Cy5^+^ cells in peripheral blood, but none were detected in spleen or bone marrow at this timepoint. This result suggested that some pDNA rapidly enters the peripheral blood from the injection site. Fluorescence microscopy of popliteal lymph nodes showed labelled DNA in the subcapsular sinus and throughout paracortical areas (data not shown), as has been described previously [19], suggesting that injected pDNA drains into the proximal lymph nodes via the afferent lymphatic vessels. In all cases, cell suspensions from unlabelled pDNA-immunised mice showed very little background staining (<0.04%). At 24 h we found pDNA-Cy5-containing cells in draining (popLN and ILN) and distal peripheral lymph nodes (Fig. 5A, bottom panel). As observed for the 1 h timepoint, the popliteal LN contained the highest percentage of positive cells (∼0.4% live cells). Although we were unable to find cell-associated pDNA in the peripheral blood at 24 h, we were able to demonstrate positive cells in both the spleen and bone marrow at this timepoint.

In other experiments, we attempted to characterise the cells associated with pDNA-Cy5 using multicolour flow cytometry. Analysis of draining and distal LNs and spleen at 24 h indicated that they were CD45/Ly5^+^ (haematopoietic), MHC Class II^+^, CD11b^+^ and mostly B220^−^, although a few B220^+^ cells were also associated with pDNA-Cy5 (Fig. 5B and Table 1). pDNA was rarely found in CD11c^high^ cells, suggesting that monocytic cells, possibly macrophages or immature monocytes (CD11b^+^, CD11c^−^) are the predominant cell type initially associated with pDNA following intramuscular DNA injection. Too few pDNA-Cy5^+^ cells were found in peripheral blood to phenotype. pDNA in bone marrow was restricted to CD45/Ly5^+^, CD11b^+^, MHC Class II^−^, which is suggestive of an immature myeloid/monocyte cell phenotype. Data presented from one experiment (*n* = 3 per group) shows that the percentage of pDNA-Cy5^+^ cells is statistically increased in both popliteal LN and spleen at 24 h (Fig. 5C). The percentage is increased in 2 out of 3 mice in the BM but does not reach statistical significance. In summary, pDNA is cell-associated in LNs draining the injection, in more distal LNs, in peripheral blood, spleen and BM, thus suggesting that pDNA is widely disseminated following intramuscular injection and hence there are multiple pathways for pDNA to reach secondary lymphoid tissue.

### 3.6 Detection of Ag and cells displaying pMHC complexes following DNA injection

We (this study), and others [1], have observed pMHC-bearing cells in peripheral lymph nodes soon after a single immunisation of soluble protein Ag, with large numbers of CD11c^+^ cells bearing pMHC complexes at 24 h post-injection. Thereafter the number of pMHC^+^ cells declines and reaches background levels by about 7 days post-immunisation (data not shown). We wanted to determine if this same strategy was sufficiently sensitive to detect pMHC^+^ cells following DNA injection where small amounts of antigen are produced *in vivo*, in contrast to bolus injection of protein Ag. We were specifically interested in both the kinetics of appearance and the anatomical distribution of pMHC complex-bearing cells following pDNA injection.

**Fig. 6:**
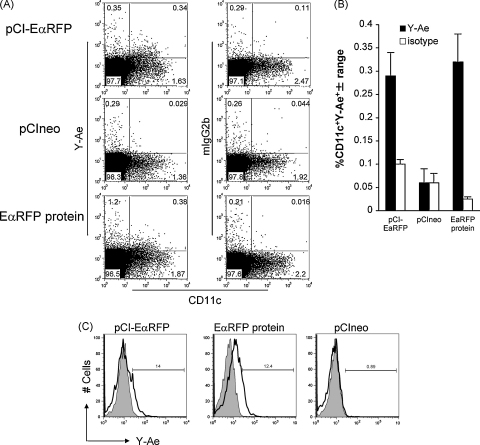
Detection of pMHC complexes in lymphoid tissue following DNA injection. (A) Flow cytometric analysis of pooled peripheral lymph nodes collected 3 days after pCI-EαRFP injection, revealed a small population of Y-Ae^+^CD11c^+^ cells (upper right quadrant), representing approximately 0.3% of live cells. pCIneo-immunised mice and isotype (mIgG2b) controls showed only background staining (0.03% and 0.11%, respectively). The proportion of Y-Ae^+^CD11c^+^ cells in pCI-EαRFP-immunised mice (i.e. 0.34%) is comparable to that seen 3 days after immunisation with EαRFP protein, i.e. several days after the peak of pMHC complex display. pCIneo-immunised mice and isotype (mIgG2b) controls showed only background staining (typically <0.1%). Results from one experiment (*n* = 2) are shown in (B) and other experiments (*n* = 3) showed a similar trend. The percentage of Y-Ae^+^CD11c^+^ cells is higher in pCI-EαRFP-immunised mice compared to both pCIneo-immunised mice and for isotype control staining. Analysis of CD11c^+^ gated cells (C) showed that approximately 14% and 12% of CD11c^+^ cells were also Y-Ae^+^ for pCI-EαRFP and EαRFP protein, respectively. Although the percentage of CD11c^+^ cells displaying pMHC was similar, the pattern of Y-Ae expression was quite different. We observed a shift in Y-Ae expression for the entire population following EαRFP protein immunisation, relative to its’ isotype control, whereas only a discrete population was positive following pCI-EαRFP injection. There was little change in Y-Ae expression following pCIneo immunisation.

Flow cytometric analysis of live cells from pooled peripheral lymph nodes collected 3 days after pCI-EαRFP injection, revealed a small population of Y-Ae^+^CD11c^+^ cells, representing 0.34% of live cells (Fig. 6A, upper right quadrants). pCIneo-immunised mice and isotype (mIgG2b) controls showed only background staining (0.03% and 0.11%, respectively). The proportion of Y-Ae^+^CD11c^+^ cells in pCI-EαRFP-immunised mice (i.e. 0.34%) is comparable to that seen 3 days after immunisation with EαRFP protein, i.e. several days after the peak of pMHC complex display. Results from one experiment (*n* = 2) are shown in Fig. 6B and other experiments (*n* = 3) showed a similar trend. The percentage of Y-Ae^+^CD11c^+^ cells is higher in pCI-EαRFP-immunised mice compared to both pCIneo-immunised mice and for isotype control staining. The percentage of Y-Ae^+^CD11c^−^ cells in pCI-EαRFP-immunised mice was no different to that observed for pCIneo-immunised mice (Fig. 6A, upper left quadrants), suggesting that the only cells that display pMHC complexes in DNA immunised mice are CD11c^+^ cells, presumably dendritic cells. This is in contrast to what we observed following EαRFP and EαGFP protein immunisation, where about 1% of live cells are Y-Ae^+^CD11c^−^ (Figs. 6A and 1B). When we gated on CD11c^+^ cells from draining lymph nodes of pCI-EαRFP- and EαRFP protein-immunised mice at day 3 following injection, we observed that approximately 14% and 12% respectively of these CD11c^+^ cells were Y-Ae^+^ (Fig. 6C). Although the percentage of CD11c^+^ cells displaying pMHC complexes was similar, the pattern of Y-Ae expression was quite different. We observed a shift in Y-Ae expression for the entire population following EαRFP protein immunisation, relative to its’ isotype control, whereas only a discrete population was positive following pCI-EαRFP injection. These cells were RFP^−^ (data not shown), suggesting that the EαRFP protein had already been processed or was below the level that we could detect by flow cytometry. There was little change in Y-Ae expression following pCIneo immunisation.

**Fig. 7:**
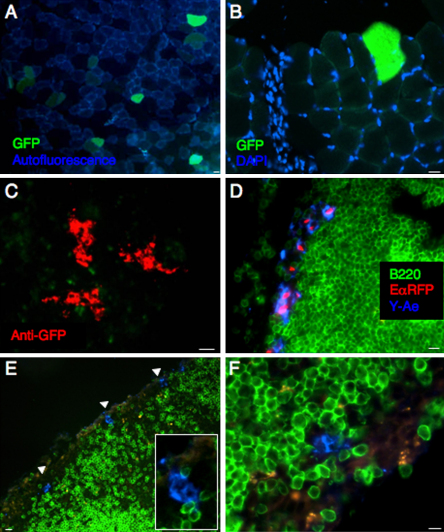
Detection of Ag and pMHC complexes in tissue sections of pDNA-immunised mice. At different times after injection of pCI-EαRFP, pCI-EαGFP or pCIneo, we collected and processed TA muscle and draining and distal lymph nodes for immunofluorescence microscopy. GFP expression was observed at the muscle injection site, 24 h after pDNA injection (A and B). GFP^+^ muscle cells were predominantly found in the vicinity of the injection site, as evidenced by the inflammatory infiltrate at the needle trajectory (B). DAPI was used in (A) and (B) to stain cell nuclei. (C) Using anti-GFP IgG, we observed small clusters of GFP protein in draining popliteal LNs at 24 h post-injection (red). (D) We observed Y-Ae^+^ cells in the subcapsular sinus of EαRFP protein-immunised mice at 24 h (blue) and (E and F) in pCI-EαRFP-immunised mice at d3 post-plasmid injection (blue). No staining was observed in pCIneo-immunised mice or using the isotype control, mIgG2b (data not shown). Scale bars show 10 μm. (For interpretation of the references to colour in this figure legend, the reader is referred to the web version of the article.)

We could detect antigen GFP expression at the muscle injection site, 24 h after pDNA injection by immunofluorescence microscopy. GFP^+^ muscle cells could be easily distinguished from the autofluorescent oxidative fibres [20] (Fig. 7A and B) and were predominantly found in the vicinity of the injection site, as evidenced by the inflammatory infiltrate at the needle trajectory (Fig. 7B). Others have reported Ag expression in non-muscle, mononuclear cells of the perimysium [21] however we were unable to confirm GFP expression in these cells. We observed small clusters of GFP^+^ cells in draining popliteal LNs at 24 h post-injection, however amplification of the GFP signal using anti-GFP Ig was required to visualise these rare cells (Fig. 7C). These results suggest pDNA-encoded Ag is in the tissue draining lymph node as early as 24 h post-injection.

As previously described for the EαGFP system, we could detect Y-Ae^+^ EαRFP^+^ cells in the subcapsular sinus (Fig. 7D) and paracortical areas of draining LNs, 24 h after EαRFP injection. However many Y-Ae^+^ cells in the T cell areas were EαRFP negative, suggesting that Ag had already been processed and hence no longer fluorescent, or that these cells contained levels of EαRFP below the limits of detection by immunofluorescence microscopy. We observed cells of a similar phenotype, Y-Ae^+^EαRFP^−^, in mice immunised with pCI-EαRFP. Three days after plasmid injection, we detected rare, sparsely distributed Y-Ae^+^EαRFP^−^ cells in the subcapsular sinus of draining inguinal lymph nodes (Fig. 7E and F). No staining was observed in pCIneo-immunised mice or using the isotype control, mIgG2b (data not shown). We were unable to conclusively demonstrate pMHC^+^ cells in the T cell areas of peripheral lymph nodes or spleen, presumably because the level of pMHC complex on these very rare cells was below the sensitivity of detection of the immunofluorescence staining protocol.

### 3.7 Eα-specific T cells accumulate and blast as early as 3 days following DNA injection

Others have shown previously that Ag dose has consequences for both the number of pMHC complexes generated and T cell activation *in vivo* and hence we were interested to know if the apparently low level pMHC we observed on CD11c^+^ cells was sufficient for T cell activation and whether the pMHC complex staining we observed 3 days after DNA injection correlated temporally with the activation of Eα-specific CD4^+^ T cells. We also wanted to establish the precise anatomical localisation and kinetics of CD4^+^ T cell activation and proliferation following intramuscular DNA injection and hence determine the relationship between pDNA distribution, pMHC^+^ cells and T cell activation. Therefore we used adoptive transfer of Eα-specific TEa T cells and kinetic analysis of activation and cell division following injection with Eα-expressing plasmids, to readout antigen presentation *in vivo*. The TEa TcR recognises the same pMHC complex as the Y-Ae mAb [12] and thus the initial activation/blastogenesis of these cells should be a good indication of the first time these cells see Ag, i.e. the precise timing of Ag presentation.

**Fig. 8:**
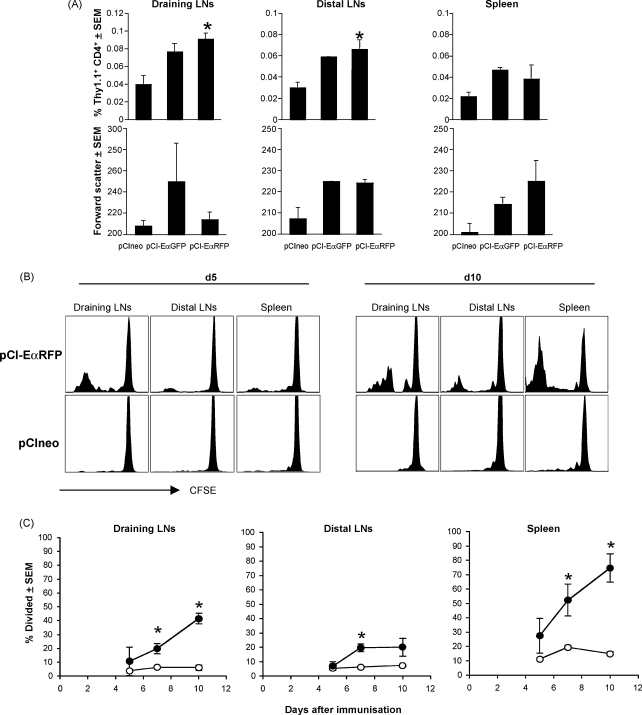
pDNA induces CD4 T cell proliferative responses early after intramuscular immunisation. To readout Ag presentation to CD4^+^ T cells *in vivo*, we transferred CFSE-labelled Eα-specific TEa T cells into B6 recipients and analysed the kinetics of T cell activation and cell division following injection with Eα-expressing plasmids. Mice that had received CFSE-labelled TEa Tg T cells 1 day previously, were immunised with pCI-EαRFP, pCI-EαGFP or the control plasmid pCIneo. Draining lymph nodes (popliteal and inguinal), distal peripheral lymph nodes (cervical, brachial and axial) and the spleen were collected at different times after immunisation (12 h–10 days) and analysed by flow cytometry for blastogenesis, clonal expansion and division of Tg lymphocytes. (A) At d3 post-immunisation we observed an increase in both the percentage and blastogenesis of Eα-specific Tg T cells in draining LNs (pooled popliteal and inguinal), distal peripheral LNs and the spleens of pCI-EαRFP- and pCI-EαGFP-immunised mice. No such increases were observed in the pCIneo group. (B and C) We observed division of TEa T cells in draining and distal lymph nodes and spleen at d5, d7 and d10 post-immunisation following injection with pCI-EαRFP but not the control plasmid pCIneo. Kinetic analysis of cell division (C) revealed that by day 5 post-pCI-EαRFP immunisation Eα-specific lymphocytes in the popLN and spleen had started to divide, and this number had increased by days 7 and 10. In contrast Eα-specific lymphocytes in distal lymph nodes has increased in number by day 7, but no further increased was observed at day 10. Cells from pCIneo-immunised mice showed only background staining at all timepoints. Error bars show standards errors and asterisk (*) indicates statistical significance compared to pCIneo-immunised group.

At early timepoints (e.g. 12 h), we observed a transient upregulation of surface CD69 in both non-Tg and Tg CD4 T cells in pCI-EαRFP- and pCIneo-immunised mice, indicative of DNA-induced non-specific activation (data not shown). However by 24 h surface CD69 had returned to control levels (data not shown). At d3 post-immunisation, we observed an increase in the percentage of Eα-specific Tg T cells in draining LNs (pooled popliteal and inguinal), distal peripheral LNs and the spleens of pCI-EαRFP- and pCI-EαGFP-immunised mice (Fig. 8A). No such increase was observed in the pCIneo group. This increase in the %Tg preceded cell division as no CFSE dye dilution was observed by d3 (data not shown). We speculate that this is indicative of retention of Eα-specific T cells or inhibition of T cell egress from the lymphoid tissues, due to stable APC-T cell interactions as we [22], and others [23] have noted in other T cell priming regimes. There was no corresponding increase in the percentage of non-Tg CD4^+^ T cells in draining LNs (Fig. 8A), distal peripheral LNs or spleen (data not shown), suggesting that the TEa accumulation we observed was Ag-driven. Concomitantly, we observed significant blastogenesis of Eα-specific T cells, in all tissues of pCI-EαRFP and pCI-EαGFP-immunised mice (Fig. 8A). No TEa blasts were found in pCIneo-immunised groups. These results are strongly suggestive of presentation Eα peptide to Eα-specific CD4^+^ T cells at d3 following plasmid vaccination and that T cells in the draining, and distal LNs and spleen have seen Ag by this time.

In order to determine if there were any differences in the kinetics of T cell activation in these anatomically distinct lymphoid tissues, we analysed cell division history using adoptive transfer of CFSE-labelled TEa T cells. By d5 we observed Eα-specific T cell division in draining lymph nodes, but little division in more distal peripheral LNs and the spleen (Fig. 8B and C). However by d10 we found TEa division in all lymphoid tissues examined, with the highest proportion of divided cells being found in the spleen. Thus although the T cell response to pDNA-encoded Ag appears to commence in the local draining lymph nodes, this is superceded by responses in the spleen. We also examined intermediate timepoints, and have never observed the multiple division peaks, typically found when using CFSE for T cell proliferation, suggesting that the Eα-specific T cells had divided in a different location and once divided had migrated to the tissues examined, or that very few naïve re-circulating T cells synchronously enter cell division, presumably due to limiting amounts of Ag. Only when they have divided more than 6 times have they accumulated sufficiently for us to detect cell division. We were unable to find evidence for Ag presentation at timepoints other than d3. These results correlate with the appearance of pMHC complexes in draining lymph nodes, hence from our data it appears that Ag presentation peaks 3 days after DNA immunisation.

## 4 Discussion

The key events in the initiation of an immune response following pDNA vaccination include cell transfection and pDNA dissemination, Ag expression in somatic and/or haematopoietic cells, Ag acquisition by APCs, presentation of peptide/MHC complexes (pMHC) to naïve T lymphocytes in secondary lymphoid tissue, development of effector and memory phenotype and function and cognate interactions between Ag-specific CD4 T cells and B cells resulting in high affinity antibody production. Despite extensive investigations demonstrating that immune responses are induced by many experimental DNA vaccines and that their character and magnitude can be readily manipulated, many of the processes noted above, related to DNA vaccines are still a “black box” with respect to the precise cell phenotypes, cell–cell interactions and anatomical and temporal aspects of the initiation and maintenance of DNA vaccine immune responses. Studies such as these are difficult because of the paucity of tools necessary to investigate these low frequency events, but crucial for the rational design and application of DNA vaccines. We have therefore applied a variety of novel tools to address these questions directly *in vivo* for the first time.

### 4.1 Anatomical distribution of pDNA

Following intramuscular injection, free and cell-associated pDNA has been found in muscle, peripheral blood [24], lymph nodes draining the injection site [19] and other sites including the bone marrow [25], minutes to months after injection [19,26–28]. Similar to others [19], we found labelled, cell-associated pDNA in the peripheral blood within 1 h of DNA injection and within cells of distal LNs, spleen and bone marrow by 24 h. We have not excluded the possibility that cells may be responsible for pDNA transport to the spleen and bone marrow, however our finding of pDNA in peripheral blood within 1 h suggests that pDNA is carried as free DNA.

Contrary to recent reports [29] we found no evidence for naïve CD4 T cell priming in the BM following pDNA injection. Our finding of pDNA-bearing cells in this site may have important consequences for both mobilisation of APC precursors from the BM into the periphery, as well as the maintenance of long-term memory following DNA vaccination. Our data suggests that CD11b^+^B220^−^MHCII^low^ cells in the BM acquire pDNA. This phenotype is consistent with monocytes or neutrophils [30] which migrate from sites of inflammation to the BM and lead to antigen presentation directly or following engulfment by another APC [30].

### 4.2 Characterisation of APC expressing pMHC

Although it is understood that DNA vaccines result in sustained Ag expression at the site of injection [31], in some cases more than 12 months [16,31–34], the exact contribution of this Ag to initiating and maintaining immune responses is far from clear. The cell types engaged in antigen production following intramuscular pDNA injection are predominantly myocytes, although direct transfection of, and antigen expression by, haematopoietic cells (including CD11b^+^ cells) at the injection site, has been reported [21,35,36]. Although it is believed that somatic cells such as myocytes serve as Ag factories, that continue to “tickle” naïve and perhaps memory cells, precisely how and when Ag gets from these Ag depots to CD4 and CD8 T cells in secondary lymphoid tissue is not clear. One of the reasons why this has been difficult to study, relates to the small amounts of Ag available for presentation at any given time which is in the nanogram range [17,34,37,38].

We demonstrated GFP expression in myocytes surrounding the injection site within 24 h of DNA injection and were able to demonstrate very rare cells containing the model Ag EαGFP in lymph nodes draining the muscle injection site at 48 h after injection (Fig. 7C) though this was at the limit of our carefully controlled detection systems. Because these cells were very rare and difficult to detect, we were unable to confirm whether they themselves had expressed the Ag, or had acquired Ag from another cell, nor could we definitively phenotype and further characterise these cells. However, their location within the LN paracortex and their dendritic appearance, suggests they may be dendritic cells and potentially able to present Ag to naïve T lymphocytes. Single cell analysis using sensitive techniques such real-time PCR may be particularly informative for determining precisely which cells express the acquired DNA and hence the contribution of direct versus cross priming for priming DNA vaccine-induced antigen presentation.

Hence the identity of the cell presenting DNA-encoded antigen to naïve T cells remains controversial and there appear to be roles for Ag presentation both by directly transfected dendritic cells and by antigen transfer from somatic cells to APCs [39,40,41].

As noted above antigen dose and persistence has significant functional consequences for the development of long-lived memory lymphocytes and hence is an important consideration for DNA vaccine design. Brief exposure to high amounts of Ag is often associated with the rapid expansion of effector CD8 T cells but limited development of long-lived memory T cells, whereas prolonged exposure to lower Ag amounts, can induce higher numbers of (central) memory cells [9,42,43]. In other studies, the precursors of long-lived memory CD4 T cells were shown to undergo lower degrees of cellular activation following their first Ag encounter, and this was a consequence of their exposure to low amounts of Ag [44]. Thus achieving the ideal balance between Ag dose, persistence and T cell activation is a very important and complex consideration for vaccines.

This led us to evaluate the minimal requirements, with respect to Ag dose and number of peptide–MHC-bearing cells necessary to elicit immune responses *in vivo* and to relate this to what we see following DNA injection. We utilised and adapted a strategy for identifying cells displaying pMHC complexes using fluorescent reporters, Eα-peptide, pMHC Ab Y-Ae and Eα-specific T cells. Itano et al. [1], reported that the induction of immune responses, following immunisation with the EαRFP protein, was characterised by two distinct waves of Ag presentation and that optimal T cell activation required both phenomena. In this previous study, EαRFP flowing into the draining LN from the injection site was processed and displayed on the surface of LN CD11c^+^ cells within minutes whereas LN migrants from the skin injection site (dermal DCs) arrived 12–24 h after immunisation. It was this second wave of pMHC^+^ cells that was essential for full CD4^+^ T cell differentiation and effector function. We observed very similar kinetics using our EαGFP fusion protein, to that reported previously and following the initial appearance of GFP^+^ and Y-Ae^+^ cells in the draining LNs at 1–4 h, these cells decreased until 12–24 h when a second wave of migrants arrived from the injection site. By 24 h we observed large numbers of Y-Ae^+^ cells, although they showed considerable heterogeneity with respect to both GFP and CD11c expression. This may reflect different states of maturation and/or different cell lineages (e.g. myeloid DC vs. pDC). Although we observed Y-Ae^+^ and GFP^+^ cells in non-draining LNs (data not shown), the low frequency of these cells highlights how Ag distribution and thus effective Ag dose, has important consequences for the location and/or duration of Ag presentation. Similarly, when we immunised with different Ag doses we observed rapid diminution of our ability to detect cell-associated Ag and pMHC complexes with decreasing Ag dose. Ag doses lower than 100 μg substantially decreased our ability to detect GFP^+^ or Y-Ae^+^ cells within both the CD11c^+^ and CD11c^low/−^ populations, however we were confident that we could detect cells from these unpurified cell suspensions down to a dose of 1 μg–100 ng. Selective enrichment of Y-Ae^+^ cells may further improve the sensitivity of these analyses.

Collectively, our results using EαGFP (and EαRFP) protein, highlight the impact of Ag dose and distribution and importance of detailed kinetic analyses for detecting rare pMHC cells *in vivo*. Nevertheless, we did detect rare pMHC^+^CD11c^+^ cells in the peripheral LNs of pDNA-immunised mice, 3 days after injection. In contrast to the clearly defined, although heterogeneous, Y-Ae^+^ cells we observed 24 h after protein injection, we did not observe a discrete population of pMHC^high^ cells, but rather an increase in Y-Ae fluorescence intensity of about 14% of CD11c^+^ cells. This was similar to what we observed 72 h after protein immunisation, when Ag was limiting. We were unable to demonstrate CD11c^+^pMHC^+^ cells in tissue sections, which was not particularly surprising as we observed only a slight increase in fluorescence intensity by flow cytometry. However, we observed dispersed Y-Ae^high^ cells in the subcapsular sinus of draining LNs, 3 days after injection of Eα-expressing plasmids. Due to the scarcity of these cells we were unable to phenotype them further, but their location in the subcapsular sinus suggests they had migrated to the LNs in afferent lymphatics or were subcapsular sinus resident macrophages [45,46].

### 4.3 Characterisation of the early T cell response

Antigen-specific T cells are transiently “enriched” in the draining lymph nodes and Ag-containing tissue sites during the early stages of an immune response due to changes in the balance between lymphocyte ingress and egress [47]. Our finding that Eα-specific T cells accumulate in peripheral LNs and spleen, 3 days after injection of Eα-expressing plasmids, suggests that these cells are involved in Ag-specific interactions with Ag-bearing APCs. This is unlikely to be simply the result of LN shut down [48–50] as the proportion of non-Tg CD4 T cells was unaltered at this timepoint (Fig. 8A). We routinely observe enlarged, hypercellular peripheral LNs between 24 and 48 h after immunisation with all plasmids, including pCIneo (data not shown), presumably due to CpG-driven non-specific inflammation, however we believe that the accumulation and/or inhibition of egress at d3 is Ag-driven and is indicative of ongoing Ag presentation. We also observed Eα-specific TEa blastogenesis at d3 and cell division by d4/d5, which is further indicative of Ag presentation occurring by d3. We were unable to find pMHC^+^ cells in the spleen, but the fact that we observed concomitant T cell accumulation and blastogenesis in draining LNs, distal LNs and spleen indicates that these things are happening at diverse locations simultaneously. T cell division in the draining LNs preceded that in the distal LNs and spleen which suggest that although T cells appear to be activated at sites distal to the tissue injection site, perhaps they do not receive sufficient stimulus, Ag dose or inflammation-driven co-stimulation at these earliest time points, to enter cell cycle. While further experiments are required to conclusively determine that cells are dividing at these sites *in situ*, and have not just migrated, the fact that pDNA reaches lymph nodes distal to the injection site and the spleen, is suggestive of Ag presentation *in situ*.

We cannot rule out Ag presentation, after d3, but we were unable to find pMHC^+^ cells after this timepoint. Increasing the sensitivity of the Y-Ae detection method may reveal a longer duration of presentation. The duration of antigenic stimulus determines the fate of naïve and effector cells, in terms of whether T cells will be activated or deleted. We know that Ag persists in the injection site and potentially the draining lymph node for many weeks, and therefore it is possible that naïve, re-circulating Ag-specific T cells may be subsequently exposed to Ag upon passage through Ag-containing lymphoid tissues, although this will depend on their precursor frequency. Whether or not these subsequently activated cells contribute to the effector or memory response is unclear. Recent evidence suggests that naïve CD4^+^ T cells that enter the immune response after the peak response, i.e. when Ag is limiting, divide less on primary response, but are better at responding upon subsequent Ag challenge, and acquire a long-lived central memory phenotype [44]. This may parallel what happens with DNA vaccines, where Ag is limiting and although primary effector responses are often weak, memory responses are usually good [11].

We have presented *in vivo*, for the first time a highly detailed description of the early events following DNA vaccination and this has considerable implications for the rational development, manipulation and application of DNA vaccination. Our data is consistent with the following scenario. Injected DNA vaccines rapidly enter the peripheral blood from the injection site but also reach lymphoid tissues directly as free DNA via the afferent lymphatics. The relatively large molecular size of pDNA probably precludes it from flowing into the conduits of LNs, and thereby LN resident DCs from sampling it directly, but rather it may be taken up by cells in the subcapsular sinus that then migrate into deeper areas of the LN such as the DC and T cell-containing interfollicular and paracortical areas. pDNA and/or expressed Ag may then be transferred from these cells to CD11c^+^ DCs for presentation to naïve T cells. Concomitantly, bloodborne DNA reaches the bone marrow and spleen where it is taken up by CD11b^+^MHCII^low^ cells (monocytes/myeloid DC precursors). The bone marrow may then act as a reservoir for cell-associated pDNA or its presence may induce the maturation and mobilisation of monocytes/myeloid DC precursors into the periphery. The observation that naïve CD4 T cells in draining and distal LNs and spleen “see” Ag simultaneously, suggests that pMHC complexes are widely distributed and the rapid dissemination of pDNA may be the reason for this. Although we were unable to precisely identify and definitively link the cells acquiring, expressing and presenting DNA-encoded Ag, due to the minute amounts of Ag involved and the rarity of these cells, they are clearly able to initiate DNA vaccine-induced immune responses.

## Figures and Tables

**Table 1:** Phenotype of pDNA-Cy5^+^ cells in different tissues 24 h after plasmid injection.

	CD45	MHCII	CD11b	CD11c	B220
LN	+	+	+	− > +	− > +
Spleen	+	+	+ > −	− > +	−
PB	+	+	ND^a^	ND	ND
BM	+	−	+	−	−

a Not determined—too few cells to phenotype further.
